# The major urinary protein gene cluster knockout mouse as a novel model for translational metabolism research

**DOI:** 10.1038/s41598-022-17195-y

**Published:** 2022-08-01

**Authors:** Sarah Greve, Gisela A. Kuhn, Mara D. Saenz-de-Juano, Adhideb Ghosh, Ferdinand von Meyenn, Katrin Giller

**Affiliations:** 1 Animal Nutrition, ETH Zurich, Universitaetstrasse 2, 8092 Zurich, Switzerland; 2 Institute for Biomechanics, ETH Zurich, Leopold-Ruzicka-Weg 4, 8093 Zurich, Switzerland; 3 Animal Physiology, ETH Zurich, Universitaetstrasse 2, 8092 Zurich, Switzerland; 4 Laboratory of Nutrition and Metabolic Epigenetics, ETH Zurich, Schorenstrasse 16, 8603 Schwerzenbach, Switzerland

**Keywords:** Fat metabolism, Homeostasis

## Abstract

Scientific evidence suggests that not only murine scent communication is regulated by major urinary proteins, but that their expression may also vary in response to metabolism via a yet unknown mechanism. Major urinary proteins are expressed mainly in the liver, showing a sexually dimorphic pattern with substantially higher expression in males. Here, we investigate the metabolic implications of a major urinary protein knockout in twelve-week-old male and female C57BL/6N mice during ad libitum feeding. Despite both sexes of major urinary protein knockout mice displayed numerically increased body weight and visceral adipose tissue proportions compared to sex-matched wildtype mice, the main genotype-specific metabolic differences were observed exclusively in males. Male major urinary protein knockout mice exhibited plasma and hepatic lipid accumulation accompanied by a hepatic transcriptome indicating an activation of lipogenesis. These findings match the higher major urinary protein expression in male compared to female wildtype mice, suggesting a more distinct reduction in energy requirements in male compared to female major urinary protein knockout mice. The observed sex-specific anabolic phenotype confirms a role of major urinary protein in metabolism and, since major urinary proteins are not expressed in humans, suggests the major urinary protein knockout mouse as a potential alternative model for translational metabolism research which needs to be further elucidated.

## Introduction

The mouse major urinary protein (Mup) family comprises at least 21 functional low molecular weight (18–19 kDa) isoforms encoded by a genomic region of 2.2 mbp on chromosome 4^1^. Due to their common ancestry, the sequences of the Mup isoforms are highly homologous^2^. The Mup belong to the group of lipocalins which are characterized by a conserved β-barrel structure, serving as transporters for a variety of hydrophobic ligands comprising pheromones, steroid hormones, retinoids, and lipids^1^. The Mup are predominantly expressed in the liver in a sexually dimorphic pattern with several times higher expression in males than in females^1^ and have been shown to be multihormonally regulated by testosterone, growth hormone, and thyroxine^3–5^. In addition to hepatic Mup expression, Mup is expressed in several secretory tissues, most notably in the submaxillary gland^6,7^, while the Mup expression occurring in other tissues is negligible^3^. Following their release into the systemic circulation, Mup are excreted via the urine where they account for approximately 90% of the total protein content in males and ensure a controlled release of pheromones from the urinary scent marks^1^. In addition to this important role in scent communication with conspecifics, an association of Mup with metabolism has also been reported. Reduced Mup expression and concomitantly lower Mup plasma concentrations were found in obese and diabetic mice^8,9^, and under conditions of decreased energy intake^10–13^. Lower levels of Mup may impair pheromone signaling and thus may contribute to the reduced reproductive activity observed during excessive energy intake as well as energy deprivation^14–16^. Both states are associated with elevated circulating free fatty acids (FFA)^17,18^, thus suggesting that FFA might act as regulatory factors for Mup synthesis^19^. In addition to being regulated by metabolism, Mup seem to be involved in regulating metabolism vice versa. Application of recombinant Mup1 to genetically obese and diet-induced obese mice improved insulin sensitivity via the suppression of the key gluconeogenic enzymes glucose-6-phosphatase (G6pc) and carbohydrate-responsive element-binding protein (Chrebp) as well as the lipogenic enzymes stearoyl-CoA desaturase-1 (Scd1) and fatty acid synthase (Fasn)^8^. Studies on the metabolic implications of Mup are scarce but those available continuously point towards a potentially crucial regulatory role of Mup in murine metabolism. It seems likely that the particularly high synthesis rate of hepatic Mup, especially in male mice^20^, requires high amounts of energy that can be saved during food scarcity by downregulation of Mup synthesis. This aligns well with previous observations^10,13^ and would decrease energy requirements to counteract energy restriction. Importantly, humans do not possess a functional Mup gene^2^ and thus lack this potential buffering system. Regarding the suggested role of Mup as a regulator of energy metabolism, the lack of Mup in humans might contribute to the recognized limitations of transferring metabolism-related research results obtained from studies in mice to the human physiology^21,22^. The implication of Mup becomes even more important when the sexually dimorphic murine Mup synthesis is considered^5^, supporting the nowadays widely acknowledged perception that ideally, mouse experiments should be performed in both sexes to obtain more valid data^23^. A Mup knockout (Mup-KO) mouse might be a better model organism for human nutrition than the commonly used mouse models with inter- and intraindividually varying expression and synthesis of the different Mup isoforms.

The present study is the first one to investigate the role of Mup in murine metabolism by deleting Mup using CRISPR/Cas9 (total Mup-KO) in male and female C57BL/6N mice. We hypothesized that (i) the presumed reduction in energy requirements by the Mup-KO results in an anabolic phenotype compared to wildtype (WT) mice, (ii) the metabolic changes mediated by the Mup-KO affect both the hepatic transcriptome and epigenome, and (iii) the effects of the Mup-KO are more pronounced in males than in females due to the higher hepatic Mup production in male compared to female WT mice. The results of the present study contribute to understanding the sex-specific metabolic implications of murine Mup and point towards the potential use of the Mup-KO mouse as an improved animal model for human metabolism research.

## Results

### The Mup gene cluster was successfully deleted to produce homozygous C57BL/6N Mup-KO mice

**Figure 1:**
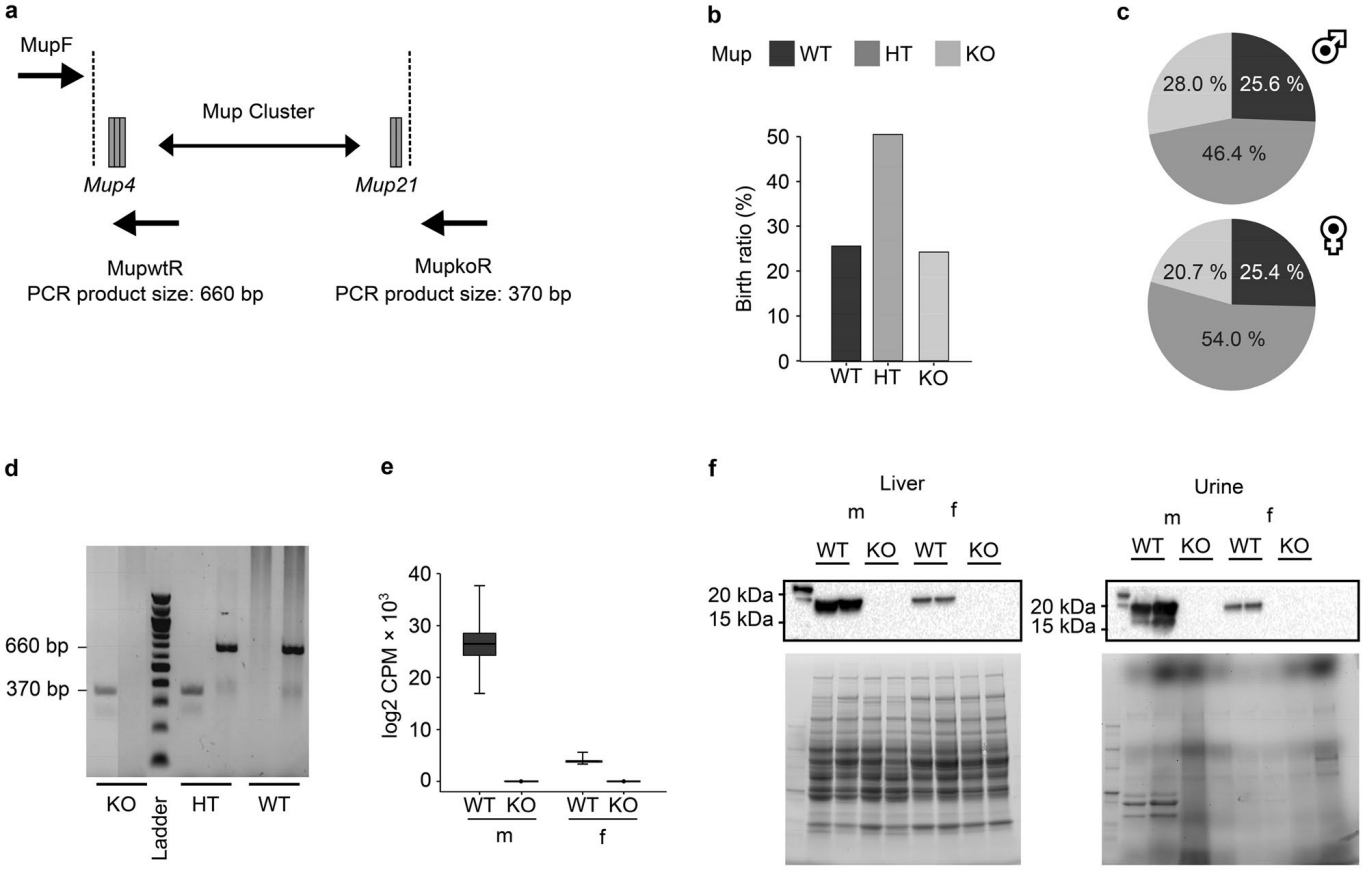
Generation and confirmation of the Mup knockout (KO) mouse model and corresponding genotype birth ratio in the colony. (**a**) Schematic representation of the Mup gene cluster on chromosome 4 with Cas9 cleavage sites (dashed lines) and primer binding sites for genotyping (single-headed black arrows). (**b**) Overall genotype birth ratio of the Mup-KO mouse line (n = 1048). (**c**) Sex-specific genotype birth ratio in male (m; n = 496) and female (f; n = 552) mice. (**d**) Representative PCR products of genotyped homozygous (KO), heterozygous (HT), and wildtype (WT) animals on an agarose gel. Primer pairs were tested separately (left lane: KO (370 bp); right lane: WT (660 bp)). (**e**) Hepatic total Mup mRNA expression levels in log2 counts per million (CPM) in male (m) and female (f) KO and WT mice quantified by RNAseq. Data are presented as Spear style boxplots, where the whiskers indicate the minimum and maximum value, and lower and upper hinges correspond to the upper and lower quartiles, and the center line to the median (mWT = 6, mKO = 5, fWT = 6, fKO = 6). (**f**) Representative Western blots (cropped; original blot membranes: Supplementary Fig. S1a) of total Mup protein levels in liver cytosolic fraction and spot urine of male and female KO and WT mice including the respective protein loading control on stain free polyacrylamide gels (cropped, original stain free polyacrylamide gel: Supplementary Fig. S1b).

The deletion of the whole Mup gene cluster was mediated by CRISPR/Cas9 using sgRNAs targeted upstream of Mup4 (sg1) and downstream of Mup21 (sg2) (Fig. 1a). The genotypes of the F2 progeny followed the expected Mendelian ratio (Fig. 1b). In WT mice, the ratio of male (m) to female (f) animals was 1:1 while for Mup-KO mice, it was 1:0.73 (Fig. 1c). The absence of Mup in the Mup-KO animals was confirmed by genotyping of genomic DNA (gDNA) from ear biopsies (Fig. 1d), hepatic Mup mRNA levels (Fig. 1e), as well as measurements of hepatic and urinary Mup protein levels (Fig. 1f). In the following text, "Mup-KO" will be referred to as "KO". When WT and KO are mentioned, this refers to the genotype independent of sex. For sex-specific comparisons, the respective sex abbreviation is included (mKO and mWT for male mice, fKO and fWT for female mice).

### The Mup-KO anabolic phenotype manifests particularly in male mice and is characterized by altered plasma and hepatic biomarkers and a moderately impaired body composition

**Figure 2:**
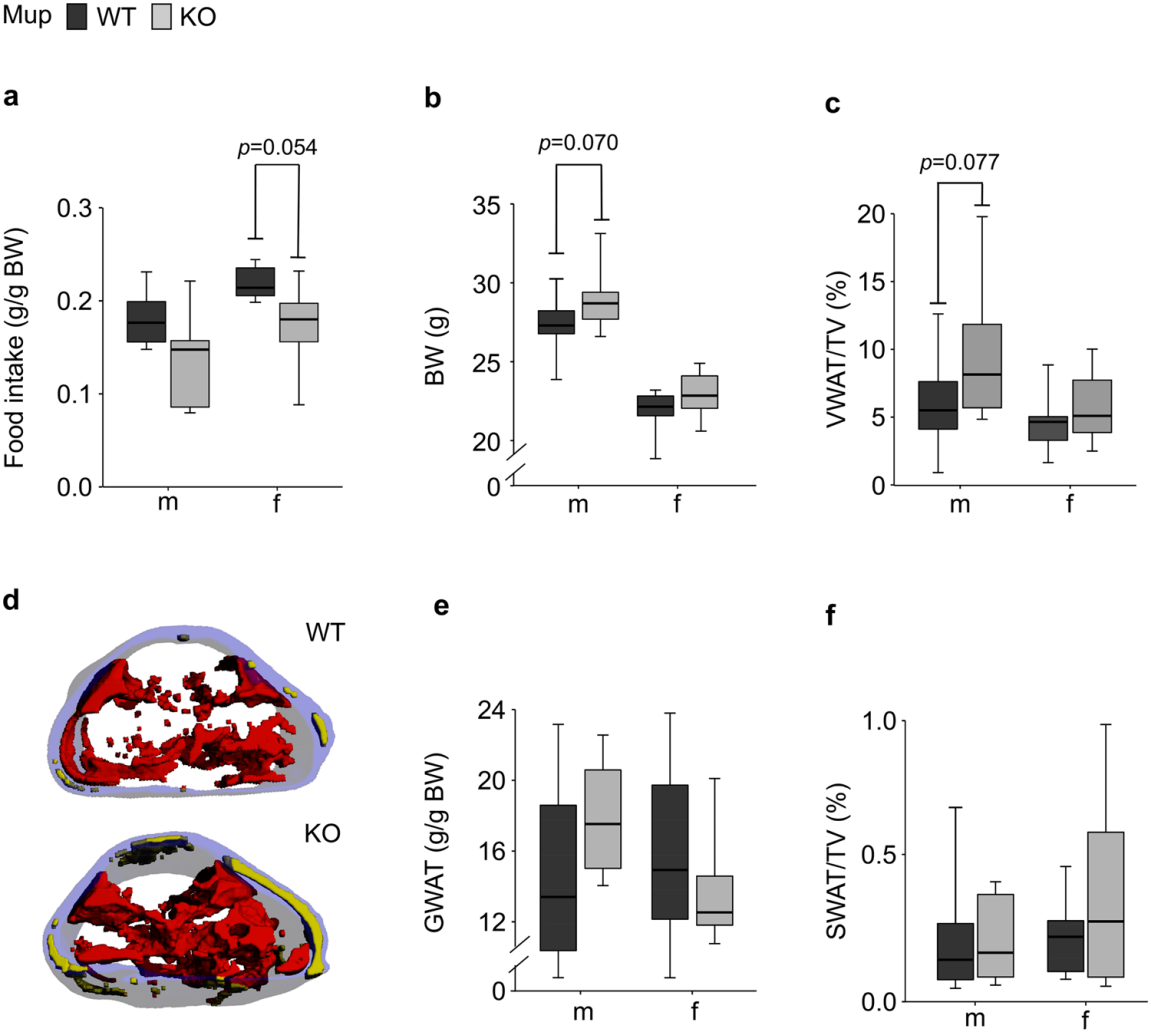
Body composition of twelve-week-old male (m) and female (f) Mup knockout (KO) mice (grey) and Mup wildtype (WT) mice (black). (**a**) Daily voluntary food intake adjusted to BW (mWT = 6, mKO = 5, fWT = 6, fKO = 6) monitored during 48 h in the Phenomaster. (**b**) Body weight (BW) according to genotype (mWT = 12, mKO = 11, fWT = 12, fKO = 12). (**c**) Proportion of visceral white adipose tissue (VWAT) volume adjusted to total volume (TV, L1–L5 region) as determined by microCT (mWT = 12, mKO = 11, fWT = 12, fKO = 12). (**d**) Representative images of adipose tissue volumes and distribution of a mWT mouse and a mKO littermate in the region of L1–L5 as determined by microCT (red: VWAT; yellow: subcutaneous white adipose tissue (SWAT)). Transparent blue edges correspond to the cutaneous area. (**e**) Gonadal white adipose tissue (GWAT) proportion adjusted to BW (all n = 6). (**f**) Proportion of SWAT volume adjusted to total volume (TV, L1–L5 region) as determined by microCT (mWT = 12, mKO = 11, fWT = 12, fKO = 12). Data are presented as Spear style boxplots, where the whiskers indicate the minimum and maximum value, and lower and upper hinges correspond to the upper and lower quartiles, and the center line to the median. *P*-values of the two-tailed t-test < 0.10 are indicated.

Between 6 and 8 weeks of age, the body weight (BW) of KO mice did not differ from that of WT mice in both sexes (averaged BW across measurements at 6, 7, and 8 weeks of age: m: 23.0 ± 0.28 (mean ± SEM) vs. 22.5 ± 0.24 g, *p* = 0.411; f: 19.1 ± 0.18 vs. 18.7 ± 0.23 g, *p* = 0.435; Supplementary Fig. S2). Furthermore, at 8 weeks of age, visceral white adipose tissue (VWAT) proportions were determined and found to be comparable in KO and WT mice in both males (5.84 ± 0.506 vs. 6.62 ± 0.713%; *p* = 0.550) and females (4.08 ± 0.300 vs. 4.37 ± 0.356%; *p* = 0.689). However, at the age of twelve weeks, and despite a numerically lower voluntary food intake in KO compared to WT mice in both sexes (m: − 22%, f: − 23%; Fig. 2a), the mKO and fKO mice had a numerically higher BW (m: + 5.4%; f: + 4.7%) than the corresponding WT mice (Fig. 2b). This was accompanied by a numerically higher proportion of VWAT (m: + 33%; f: + 21%; Fig. 2c,d) in mKO and fKO compared to the respective WT mice. Gonadal white adipose tissue (GWAT) proportion was also numerically higher in mKO compared to mWT mice while it was similar in fKO and fWT mice (Fig. 2e). The proportion of subcutaneous white adipose tissue (SWAT; Fig. 2d,f) was comparable in KO and WT mice of both sexes.

**Figure 3:**
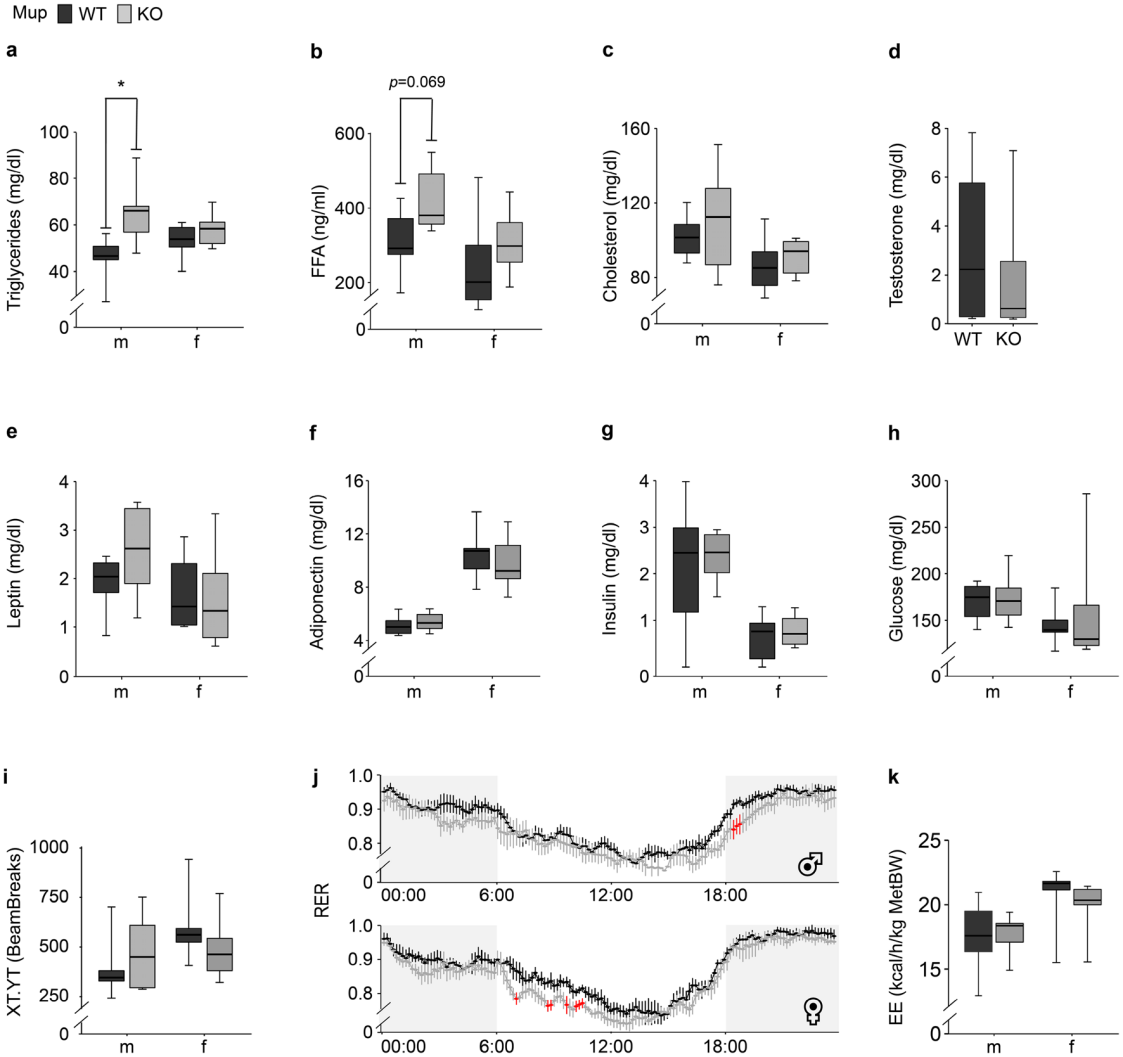
Metabolic characterization of twelve-week-old male (m) and female (f) Mup knockout (KO) mice (grey) and Mup wildtype (WT) mice (black). (**a**–**c**) Plasma concentrations of lipid metabolic biomarkers: (**a**) triglycerides, (**b**) free fatty acids (FFA) and (**c**) cholesterol. (**d**) Plasma testosterone concentration of mWT and mKO mice. (**e**,**f**) Plasma concentrations of lipid metabolism related hormones: (**e**) leptin, (**f**) adiponectin. (**g**,**h**) Plasma concentration of glucose metabolic biomarkers: (**g**) insulin, (**h**) glucose (**a**,**c**–**h**: n = 6; **b**: mWT = 6, mKO = 5, fWT = 6, fKO = 4). (**i**) Mean total 48 h activity (mWT = 6, mKO = 5, fWT = 6, fKO = 6) detected via infrared light beam breaks. (**j**) Mean circadian respiratory exchange ratio (RER) per sex according to genotype monitored over 48 h in the Phenomaster (mWT = 6, mKO = 5, fWT = 6, fKO = 6). Light (white) and dark phase (grey) is indicated. Data was adjusted to metabolic BW (lean mass + 0.2 × fat mass). Results of two-tailed t-test at specific time point: *p* < 0.05 (red). (**k**) Mean hourly energy expenditure (EE) per group (mWT = 6, mKO = 5, fWT = 6, fKO = 6) monitored over a period of 48 h and normalized to the metabolic BW (MetBW). Data (**a**–**i**,**k**) are presented as Spear style boxplots, where the whiskers indicate the minimum and maximum value, lower and upper hinges correspond to the upper and lower quartiles, and center line to the median. Results of (**j**) are presented as mean ± SEM. In (**j**) a linear model additionally accounting for repeated measurements was applied to calculate the KO effect. *P*-values of the two-tailed t-test < 0.10 are indicated. *p < 0.05 in two-tailed t-test.

Plasma concentration of triglycerides (TG) was significantly higher in mKO mice compared to mWT mice but this difference was not observed in female mice (Fig. 3a). The FFA plasma concentration was numerically higher in KO compared to WT mice in both sexes (m: + 37.3%, f: + 21.7%; Fig. 3b). The KO did not affect plasma cholesterol (Fig. 3c). Male plasma testosterone levels did not significantly differ between mKO and mWT mice (Fig. 3d). Leptin plasma concentrations were numerically higher by 35% in mKO compared to mWT mice, while no such numerical difference was observed in females (Fig. 3e). Independent of sex, adiponectin plasma concentrations (Fig. 3f) as well as the ratio of leptin:adiponectin (m: 0.47 ± 0.069 vs. 0.36 ± 0.038, *p* = 0.214; f: 0.15 ± 0.029 vs. 0.18 ± 0.050, *p* = 0.650) did not differ between KO and WT mice. Likewise, plasma insulin and glucose concentrations (Fig. 3g,h) as well as physical activity (Fig. 3i) did not differ between KO and WT mice. Automated phenotyping measurements revealed a numerically lower circadian respiratory exchange ratio (RER) in the KO compared to WT mice throughout the day, reaching significance at several time points in both sexes (Fig. 3j). The distribution of significant RER differences in male and female mice differed across the day. The RER differences between mKO and mWT occurred in the early dark phase (6 p.m–7 p.m.) while those between fKO and fWT mice were observed exclusively in the light phase (7 a.m.–11 a.m.). Oxygen uptake (VO_2_; Supplementary Fig. S3a), carbon dioxide production (VCO_2_; Supplementary Fig. S3b), and consequently energy expenditure (EE; Fig. 3k) did not differ between KO and WT mice.

### Male but not female Mup-KO mice display differential hepatic gene expression related to lipid metabolism compared to the respective WT mice

**Figure 4:**
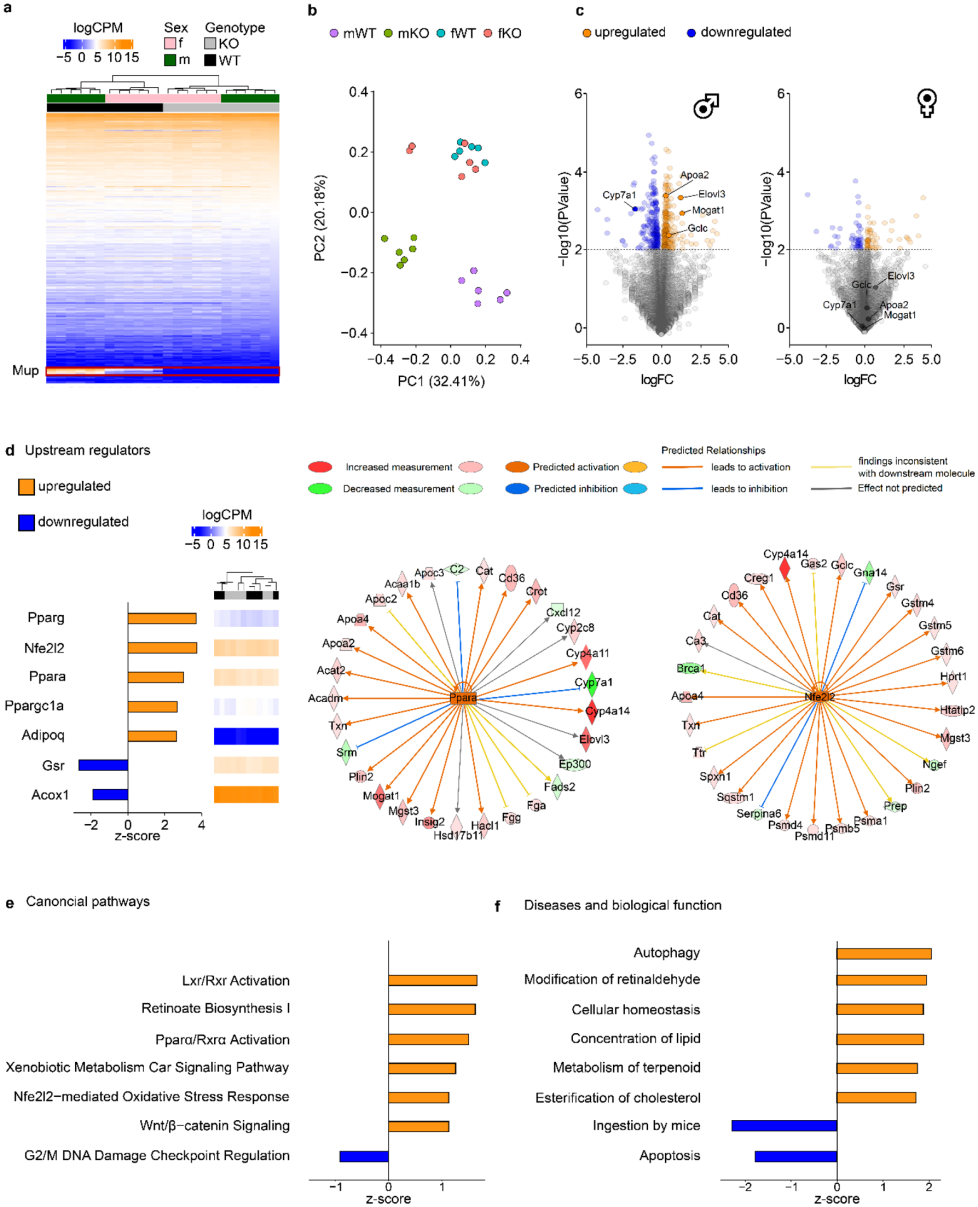
Hepatic gene expression in Mup knockout (KO) mice. (**a**) Heatmap indicating the gene expression in log_2_ counts per million (logCPM) of differentially expressed genes (DEG; n = 571; *p* < 0.01) in male (m) and female (f) KO mice compared to respective wildtype (WT) mice. Color gradient corresponds to sample-individual gene expression level (blue: low gene expression, orange: high gene expression). The Mup gene family is highlighted (red box). (**b**) PCA plot based on identified DEG among experimental groups (mWT: purple, mKO: green, fWT: blue, fKO: red). (**c**) Volcano plot of differential gene expression analysis in male and female mice. Dotted line represents cut-off for DEG identification. Key lipogenic regulated genes in mKO mice are highlighted. (**d**) Selected upstream regulators involved in lipid metabolism and oxidative stress predicted by Ingenuity Pathway analysis (IPA) based on the mKO DEG data set. Gene expression (logCPM) of upstream regulators among experimental groups is indicated in the heatmap. The IPA upstream regulator prediction of peroxisome proliferator-activated receptor alpha (Pparα) and nuclear factor, erythroid-derived 2-like 2 (Nfe2l2) including predicted relationships with identified DEG among mKO vs. mWT mice is indicated in the circle plots. Color gradient of DEG represents differential gene expression level (red: upregulated, green: downregulated). Arrows correspond to predicted IPA consensus (orange: activation, blue: inhibition, yellow: inconsistent, grey: unpredicted). (**e**,**f**) The most affected (**e**) canonical pathways and (**f**) diseases and biological function networks of identified DEG in mKO vs. mWT comparisons as predicted by IPA. The bar plots (**d**–**f**) are based on the activation z-score, consistent with the particular activation state (activated: orange, inhibited: blue). In (**c**–**f**), the Mup gene family was excluded from the DEG data set for data representation and analysis. (**a–f**) all n = 6.

**Figure 5:**
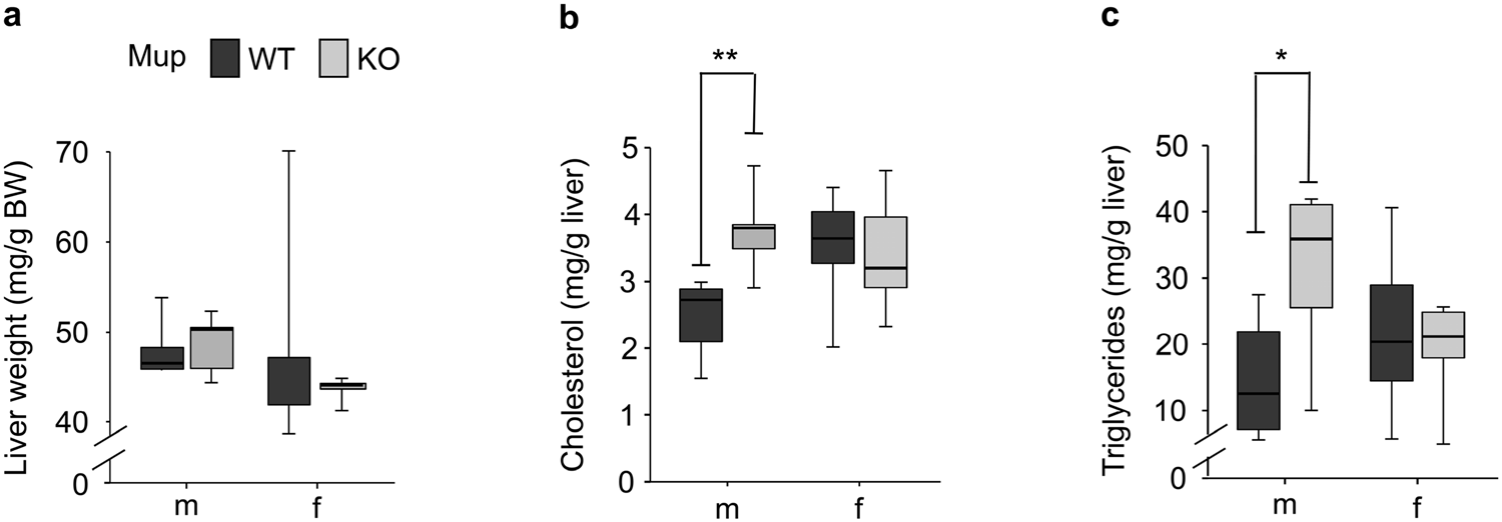
Liver weight and composition of Mup knockout (KO) mice. (**a**) Liver weight normalized to body weight (BW). (**b**,**c**) Hepatic concentrations of (**b**) cholesterol and (**c**) triglycerides in mKO and fKO mice (grey) and respective WT mice (black). The Spear style boxplot whiskers indicate the minimum and maximum value, lower and upper hinges correspond to the upper and lower quartiles, and the center line to the median. Significances (*p < 0.05, **p < 0.01) of the two-tailed t-test are indicated. (**a**–**c**) all n = 6.

A hepatic RNAseq analysis of KO and WT mice was performed to explore the formerly described anabolic changes on a molecular level. Of the 16,267 genes expressed in the liver of KO and WT mice, 461 differentially expressed genes (DEG) were found in mKO and 137 DEG were identified in fKO animals compared to mWT and fWT mice, respectively (Fig. 4a, Dataset S1). The DEG in mKO mice may explain the different phenotypes observed in mKO and mWT mice as principal component analysis of gene expression data discriminates between mKO and mWT but not between fKO and fWT animals (Fig. 4b). Overlapping downregulated DEG between mKO and fKO mice were limited to the deleted Mup gene cluster (Mup1–Mup22) whereas interleukin-1 receptor accessory protein (Il1rap) and Spry domain-containing protein 7 (Spryd7) were the only upregulated DEG overlapping between males and females. Also, DEG analysis revealed sex-specific unique gene expression patterns with 203 uniquely down- and 234 uniquely upregulated genes in mKO mice and 42 uniquely down- and 69 uniquely upregulated genes in fKO mice (Fig. 4c). Monoacylglycerol O-acyltransferase 1 (Mogat1), apolipoprotein A II (Apoa2), elongation of very long chain fatty acids protein 3 (Elovl3), and glutamate-cysteine ligase catalytic subunit (Gclc) were identified among the top upregulated DEG, while cholesterol 7 alpha-hydroxylase (Cyp7a1) was among the top downregulated DEG in the mKO vs. mWT comparison. None of these genes were significantly regulated between fKO and fWT mice (Fig. 4c). Peroxisome proliferator-activated receptor alpha (Pparα) and nuclear factor, erythroid-derived 2-like 2 (Nfe2l2) were observed as the most relevant hepatic upstream regulators activated in mKO mice (Fig. 4d). Of the 29 identified DEG associated with Pparα signaling in mKO mice, the differential regulation of 19 genes in the data set was in accordance with the curated IPA prediction based on the literature. Similarly, of the 29 identified DEG associated with Nfe2l2 signaling in mKO mice, the differential regulation of 23 genes in the data set was in accordance with the curated IPA prediction based on literature. Neither Pparα nor Nfe2l2 was regulated in fKO mice. Consequently, multiple canonical pathways related to Pparα and Nfe2l2 action were induced in mKO but not in fKO mice compared to mWT and fWT mice, respectively (Fig. 4e). In line with the predicted canonical pathways, activation of functional gene networks associated with lipid metabolism was indicated in mKO mice (Fig. 4f). Subsequent analysis of liver proportion to BW and hepatic lipid composition intended to validate the results of functional enrichment analysis of DEG. Despite similar liver proportions (Fig. 5a), hepatic cholesterol (Fig. 5b) and triglyceride (Fig. 5c) concentrations were significantly higher in mKO compared to mWT mice.

### DNA promoter methylation is unlikely to play a role in differential gene expression of mKO mice

**Figure 6:**
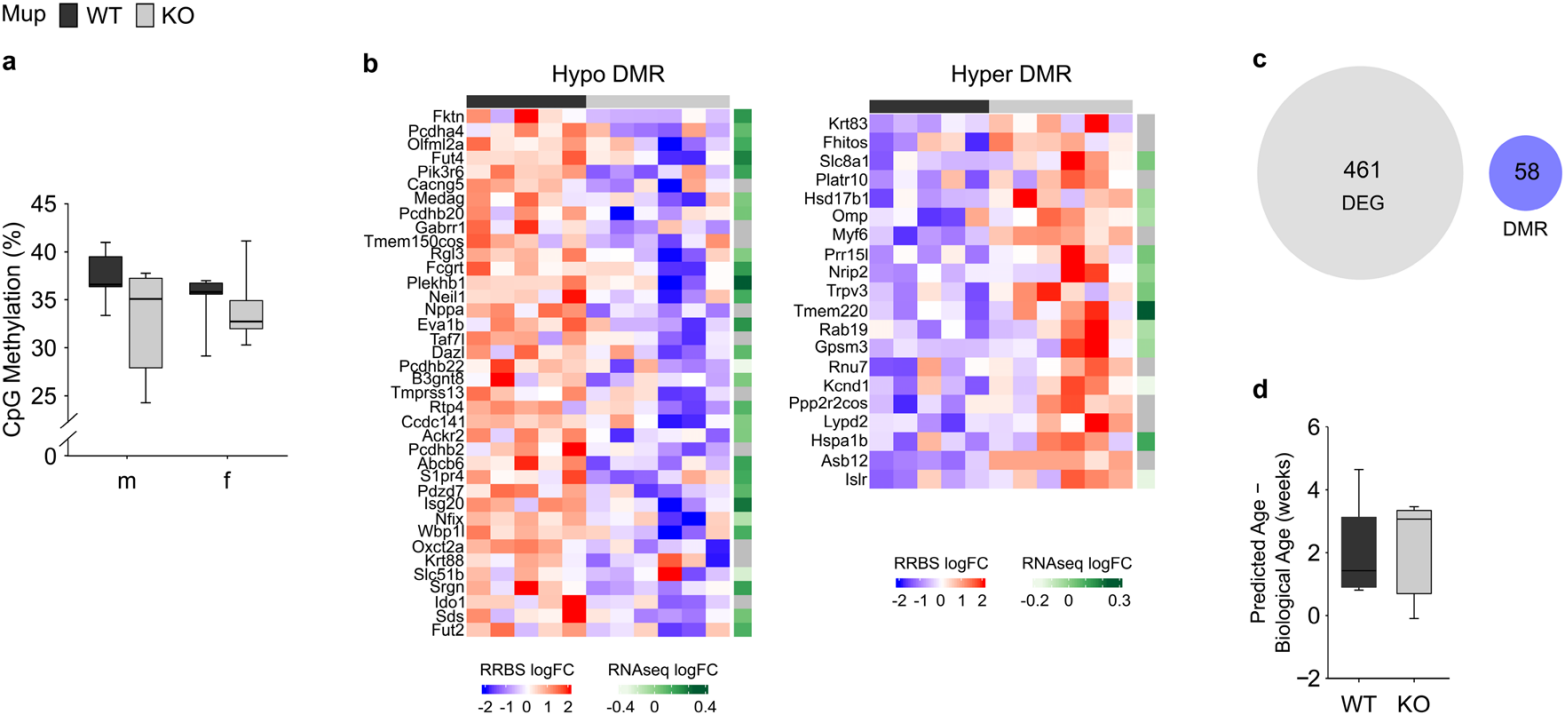
Differential hepatic DNA-methylation in Mup knockout (KO) mice and integration with transcriptomic data. (**a**) Average CpG methylation level distribution of male (m) and female (f) KO mice (grey) and their wildtype (WT) counterparts (black). (**b**) Heatmap showing the significant (*p* < 0.05) hypo- and hypermethylated differentially methylated regions (DMR) with > 5% methylation difference in male mice. Color gradient corresponds to individual DMR methylation status (red: hypermethylated, blue: hypomethylated) per sample and corresponding gene expression level retrieved from KO vs. WT differential gene expression analysis (green: upregulated, grey: downregulated). (**c**) Venn diagram showing no overlap between DMR and differentially expressed genes (DEG; *p* < 0.01). (**d**) Residual of predicted and biological age among mKO and mWT mice. Data (**a**) and (**d**) are presented as Spear style boxplots, where the whiskers indicate the minimum and maximum value, lower and upper hinges correspond to the upper and lower quartiles, and the center line to the median. (**a**) mWT = 6, mKO = 5, fWT = 6, fKO = 6; (**b**–**d**) mWT = 6, mKO = 5.

Reduced representation bisulfite sequencing (RRBS) in liver tissue showed no significant difference in overall DNA methylation between KO and WT animals (Fig. 6a). Differential methylation analysis identified 58 (20 hypermethylated, 38 hypomethylated) differentially methylated regions (DMR) in mKO mice (Fig. 6b, Dataset S2) and 71 DMR (55 hypermethylated, 16 hypomethylated) in fKO mice (Supplementary Fig. S4, Dataset S2) compared to their respective WT counterparts. In mKO mice, hypomethylated DMR were mostly correlated with increases in gene expression, while genes with promoters gaining methylation were repressed but some also activated (Fig. 6b). In fKO mice, the correlations between DNA methylation changes and gene expression were less well defined (Supplementary Fig. S4).

Next, the contribution of DNA methylation to differential gene expression in the KO compared to the WT mice was investigated. The analysis, that was focused on mKO mice, since only these showed differential hepatic gene expression (Fig. 4), revealed no overlap between identified DMR and DEG (Fig. 6c). Finally, the epigenetic age^24^ did not differ between mKO and mWT mice (Fig. 6d). Both mWT and mKO mice were predicted to be older than their actual biological age at sacrifice based on their methylation pattern.

## Discussion

To contribute to an improved understanding of the metabolic implications of Mup, we successfully established a CRISPR/Cas9 mediated KO mouse model on a C57BL/6N background as confirmed by the absence of Mup in liver and urine of mKO and fKO mice. The results of the present study verify for the first time that the sexually dimorphic expression of Mup does not only play a role in scent communication^25,26^ but also in metabolism of mice as previously suggested in the literature^8,9^.

At ad libitum access to standard chow, twelve-week-old mKO mice developed an anabolic phenotype, indicated by higher plasma TG concentrations compared to mWT mice and simultaneous accumulation of TG and cholesterol in the liver. These observations indicate an energy surplus in mKO compared to mWT mice that was clearly not caused by higher feed intake. The numerically higher BW and VWAT proportions further support the assumption of an energy surplus in mKO compared to mWT mice. Energy expenditure and activity levels were similar in mKO compared to mWT mice. In fKO mice, despite numerically increased BW, VWAT proportion, and plasma FFA concentration, the metabolic phenotype was similar compared to fWT mice.

The herein reported lack of an effect of the KO on energy expenditure and activity levels seems to contrast with the findings of *Hui *et al*. *who reported increased energy expenditure and locomotor activity in mice following Mup administration^9^. However, given the different ways of mouse model generation (KO of the Mup gene cluster in the present study versus gain of function by continuous recombinant Mup administration in metabolically impaired mice by *Hui *et al.^9^), the findings of the two studies are not necessarily contradicting.

A potentially important aspect worth considering might be an effect of the lacking Mup expression in KO mice during the early stages of ontogenesis. Given the assumed impact of Mup on metabolism, effects on the organism's development by the lack of Mup expression seem likely. Potentially, pre- and early postnatal testosterone and growth hormone surges might induce a transient Mup expression in WT mice with yet unknown effects on development that would be absent in KO mice.

Considering the formerly described metabolic cost of hepatic Mup production, the deletion of the Mup gene cluster and subsequent absence of Mup gene expression and protein synthesis in the present study likely lowered the energy requirements of KO animals^27^. Despite its high inter- and intraindividual variablity^5,6^, hepatic Mup protein synthesis represents a substantial amount of protein expression in the liver of WT animals^1^. Indeed, indirect calorimetry data revealed a slightly lower metabolic rate in KO compared to WT mice in both sexes, indicated by a numerically lower RER. However, genotype-dependent differences in energy expenditure were observed in none of the sexes, although mKO mice accumulated ≈ 1.2 g and fKO mice ≈0.4 g more VWAT during four weeks of ad libitum feeding than the respective WT mice. Given the similar BW of KO and WT mice per sex until the onset of significant Mup production at eight weeks of age^28^, we conclude that the metabolic differences between KO and WT mice observed in the present study manifested during only about four weeks of differential Mup expression and thus occurred concomitantly to ≈ 0.3 g of weekly VWAT gain in mKO mice. In fKO mice, the weekly surplus in VWAT gain compared to fWT mice was lower (≈0.1 g). According to Butler & Kozak^29^, the discriminatory capacity of indirect calorimetry requires a weekly fat accumulation of at least 0.5 g at a daily energy expenditure of 50–60 kJ to detect differences in energy expenditure between two experimental groups. The slightly lower energy expenditure (45 kJ/day) in combination with the lower weekly VWAT gain (0.1–0.3 g/week as described above) in KO versus WT mice in the present study may thus explain the lack of significant differences between KO and WT mice in terms of energy expenditure. Since WT mice did not accumulate VWAT between eight and twelve weeks of age, the Mup protein synthesis in WT mice in comparison to KO mice likely accounts at least for part of the genotype-related differences in energy consumption.

The numerically higher BW and VWAT in KO compared to WT mice of both sexes in combination with the numerically lower food intake in KO mice indicate that KO mice failed to completely adjust their food intake to maintain energy homeostasis. Consequently, excess energy intake led to the observed anabolic adaptations in KO compared to WT animals. The energy surplus is unlikely to be fully explained by the mere energy requirements for Mup synthesis. This suggests that not only an energy surplus but also an additional influence of Mup on metabolism may have contributed to the observed onset of VWAT accumulation in the KO mice.

In contrast to the numerically higher VWAT and GWAT proportions, SWAT was not significantly affected by the KO in both male and female mice. Previous research showed that during weight gain, VWAT typically is the main fat depot involved in adipose tissue remodeling and energy storage, while SWAT remains unaffected^30^. This mechanism was exclusively observed in male mice while in female mice, adipose tissue remodeling occurred in VWAT and SWAT in equal but non-significant proportions^31^. Even though numerical VWAT accumulation occurred in KO mice of both sexes in the present study, VWAT volume differed to a greater extend in mKO (+ 33%) compared to fKO mice (+ 21%).

The VWAT is not only a storage tissue but also a secretory endocrine organ^32^. Circulating FFA and leptin concentrations correlate with VWAT proportions^32,33^. Therefore, an increased proportion of VWAT enhances the liberation of FFA and leptin. The numerically higher VWAT proportion in KO compared to WT mice of the present study represents only a moderate weight gain since the BW of mKO mice was lower compared to leptin resistant and high-fat diet fed animals with a reported BW of ~ 50 g^8,9^. This likely explains the only numerically higher plasma FFA and plasma leptin concentrations observed in mKO compared to mWT mice. Since FFA and leptin are involved in appetite regulation and energy homeostasis^34–36^, their numerical differences in plasma concentrations also reflect the slight and thus physiological adaptions in food intake observed in both male and female KO compared to WT mice. The similar plasma concentrations of cholesterol, glucose, and insulin in both genotypes indicate that an obesity-induced insulin-resistance was not (yet) present in the KO mice.

In accordance with former reports, Mup protein was more abundant in liver and urine of mWT compared to fWT mice^3,6,37^. Consequently, the decrease in energy requirements caused by the KO was more pronounced in mKO than in fKO mice compared to their respective WT counterparts. A proportionately larger difference in energy requirements in mKO mice may explain why some of the effects (significantly higher plasma TG, hepatic TG, and hepatic cholesterol concentration) were observed in mKO but not in fKO mice compared to the respective WT mice.

In line with the observed anabolic changes, particularly among mKO mice, the hepatic transcriptome revealed an enrichment of pathways related to lipid metabolism in mKO but not in fKO mice compared to the respective WT animals. Accordingly, Pparα, which mediates energy homeostasis and is activated by circulating FFA^38^, was predicted as upstream regulator of multiple DEG in mKO mice. The observed hepatic lipid accumulation in mKO compared to mWT mice is also in line with the differential gene expression related to TG synthesis as well as lipoprotein transport and turnover observed in mKO mice in the present study. Differential gene expression of interest in this context was the upregulation of Elovl3 (involved in TG synthesis)^39^ and Apoa2 (a lipoprotein with proatherogenic properties by decreasing lipoprotein clearance)^40^, as well as the downregulation of Cyp7a1 (rate limiting enzyme of bile acid synthesis and consequently hepatic cholesterol catabolism)^41^. In contrast to the formerly reported effect of Mup administration on the regulation of key lipogenic genes such as Scd1 and Fasn as well as glucogenic genes (G6pc, Chrebp), the expression of these genes was not affected in the KO mice of the present study^8^. However, experimental setup and therefore the respective outcomes of the study by *Zhou* et al.^8^ and the present study are not entirely comparable as already outlined above.

Hepatic lipid accumulation is related to increased levels of reactive oxygen species (ROS) originating from enhanced lipid peroxidation^42^. The ROS induce antioxidant defense mechanisms in the hepatocytes via the activation of Nfe2l2 and, consequently, Nfe2l2-regulated antioxidant enzymes^42^. As for Pparα, the activation of Nfe2l2 compared to WT mice was exclusively observed in mKO mice and may indicate a mechanism to cope with early developing hepatic damage^43^ caused by the KO mediated hepatic lipid accumulation.

Regulation of gene expression is partially under control of epigenetic mechanisms such as DNA-methylation of promoter regions. Methylation affects the accessibility of DNA for transcription factors with promoter regions of transcribed genes usually being hypomethylated^44^. Indeed, the identified hypomethylated DMR indicated gene activation, but effects were weak and did not reach the pre-defined cut-off required for a true DEG in differential expression analysis. Similarly, hypermethylation did not lead to significant changes in gene expression. Consequently, none of the identified DEG correlated with DNA-methylation of promoter regions in the present study, implicating that the DEG were regulated by mechanisms other than promoter DNA-methylation. Epigenetic reprogramming such as DNA-methylation is a dynamic process and a hallmark of aging in humans and mice^45^. Aging can be predicted by individual methylation pattern of selected age associated CpG^24^. In our study, mKO and mWT mice did not differ in methylation-based age prediction, suggesting a genotype-independent aging rate at the age of twelve weeks.

In line with the predicted pathway activation from transcriptome data of the present study, a higher TG and cholesterol deposition was detected in the liver of mKO but not fKO mice compared to sex-matched WT mice. The combined occurrence of hepatic TG and cholesterol accumulation with differential gene expression in mKO but not in fKO mice indicates a sexually dimorphic role of Mup in metabolism, thus matching their likewise sexually dimorphic expression^3^. Even though hepatic metabolism was affected by the KO, the unaffected liver proportion and plasma cholesterol level as well as the similar hepatic DNA methylation indicate a rather moderate metabolic effect of the KO in twelve-week-old mice. Wildtype mice start to produce substantial amounts of hepatic Mup protein at the age of eight weeks and continue throughout adulthood^1,28^. Thus, it is likely that the observed anabolic changes would become more pronounced with increasing age. Overall, metabolic effects of the KO were moderate in the young mice from the present study, and we postulate that the magnitude of effects would increase with increasing animal age.

With regard to the observed sexually dimorphic effects of the KO it needs to be considered that Mup expression has been reported to also rely on hormonal and social status^5^. This might partially limit the suitability of WT mice as reliable control group, which however raises the question how an ideal control group for the KO mouse model could look like. A stable Mup expression is not achievable since the mechanisms involved in the regulation of Mup expression are not fully elucidated^5^. A stable expression would also not reflect natural, physiological conditions. Controlled continuous administration of Mup as performed by *Hui* et al.^9^ could normalize circulating Mup levels but would avoid the energy consumption necessary for Mup production^9^. These considerations stress the relevance of our KO mouse model for translational research since it removes the variability of Mup expression that all other mouse models currently in use clearly exhibit due to the highly variable intra- and interindividual Mup expression as also observed in the WT mice of the present study. The interindividual variability in Mup expression was more pronounced in male than in female WT mice. Still, the magnitude of the difference in Mup expression between KO and WT mice was many times higher than the interindividual variability in Mup expression of different WT mice as determined by RNAseq analysis. Consequently, we consider both male and female WT mice suitable controls for the respective KO animals used in the present study.

In summary, hypothesis (i): “the presumed reduction in energy requirements by the KO results in an anabolic phenotype compared to WT mice" was partially confirmed by the observed significant accumulation of hepatic lipids and the numerically higher BW and VWAT proportion in mKO compared to mWT animals. Also hypothesis (ii): “the metabolic changes mediated by KO affect both the hepatic epigenome and transcriptome” was only partially confirmed as the clear adaptations in hepatic gene expression were not related to differential promoter DNA methylation. Further epigenetic modifications potentially contributing to the observed gene expression differences between mKO and mWT mice should be investigated in the future. In this context, histone modifications and in particular the transcriptionally active H3K9ac might be of interest. The H3K9ac has previously been described as a regulator of Mup5 expression^10^ and is furthermore involved in hepatic lipid accumulation^46^. The absence of the Mup gene cluster potentially leads to a shift of H3K9ac from Mup promoter regions to lipid metabolism and oxidative stress-related gene promoters stimulating the anabolic hepatic phenotype observed in mKO. In addition, a significant upregulation of pathways involved in lipid metabolism accompanied by a higher hepatic lipid deposition was observed exclusively in mKO mice, thus confirming hypothesis (iii): “the effects of the KO are more pronounced in males than in females due to the higher hepatic Mup production in male compared to female mice”. Given the higher expression of Mup in the liver of male compared to female WT mice, we conclude that Mup deletion leads to energy excess in young mice and predominantly in males. The effects might become even more pronounced with a prolonged study period.

The continuous Mup expression and conferring energy cost throughout lifespan may have implications especially for the outcome of feeding studies with a restricted food regime and long-term studies conducted in WT mice. In rodents, dietary restriction (DR) mediates an extension of life- and healthspan^47–49^. It has previously been reported that DR (− 25% compared to ad libitum control) abolishes Mup production in male mice^10^. Considering that a lack of Mup decreases energy requirements, DR in WT mice is partly compensated for by discontinuing the Mup synthesis. Thus, DR studies in mice likely overestimate the magnitude of the applied reduced energy intake and thus, the transferability of life- and healthspan effects to organisms not expressing any Mup such as humans. This is especially true for male mice because of their several times higher Mup production compared to female mice^1^. In accordance with other studies, our results therefore clearly demonstrate that the inclusion of both sexes is compulsory to conduct scientifically reliable metabolic studies^50–52^. Given the high energy requirements for Mup synthesis, we postulate that results obtained from nutrition studies in the KO mouse might be better transferrable to humans because Mup synthesis, that can in part compensate for differential nutrient supply, is removed. The potential superiority of the KO mouse as an obesity model e.g., compared to mice on a high fat diet, needs to be further elucidated in studies with an extended feeding period where an obesity phenotype may likely develop on ad libitum feeding of standard chow. With regard to previous reports that have shown inhibition of Mup expression by aging^53^, fasting^8,9^, dietary restriction^10^, overfeeding, and obesity^8,9^, studying these conditions in KO mice is expected to further improve the understanding of the metabolic implications of Mup in mice. Furthermore, the KO mouse model has recently been suggested as a useful model for human urinalysis^54^. In the KO mice, the urinary protein status was comparable to that of humans and eliminated the Mup-related masking of other urinary proteins. Regarding translational research, we presume that the absence of Mup is not limited to urinalysis but, based on the present study results, may render mice, particularly males, metabolically more similar to humans and also removes part of the inter- and intraindividual variation otherwise induced by variable Mup production in terms of pattern and amount of Mup synthesis. On standard chow, WT mice have been shown to greatly differ in cholesterol and bile acid metabolism while exhibiting rather low cholesterol levels and high bile acid levels in comparison to humans^55^. This is due to the fundamentally different regulation of cholesterol metabolism in mice and humans^56^. Mice control their plasma cholesterol concentrations by a feed-forward mechanism which is manifested in the liver. In response to hepatic cholesterol accumulation (oxysterols), bile acid synthesis is increased which in turn promotes cholesterol excretion. On the molecular level, this mechanism is triggered by activation of the liver X receptor (Lxr) and downstream Cyp7a1^57^. In humans, however, Cyp7a1 does not represent a target of Lxr and thus the feed-forward regulation of cholesterol metabolism is not conserved^56,57^. In line with the metabolic regulation observed in humans, the mKO mice, despite their higher hepatic cholesterol accumulation, showed a downregulation of hepatic Cyp7a1 expression and may thus represent a more humanized liver phenotype compared to WT mice. In conclusion, we believe that the KO mouse has the potential to be used as an improved model to address multiple concerns in translational metabolism research which should be further elucidated.

## Material and methods

All experimental procedures involving animals were approved by the Cantonal Veterinary office of Zurich, Switzerland (license number ZH043/17) and performed in accordance with the relevant guidelines and regulations. The study is reported in compliance with the ARRIVE guidelines.

### Animals and housing

The KO mouse model was generated on a C57BL/6N background by using the CRISPR/Cas9 system as previously described^58^. In brief, a mixture of 1.2 µM Cas9, 0.6 µM sg1 (targeting upstream of Mup4), and 0.6 µM sg2 (targeting downstream of Mup21) RNAs (all obtained from Integrated DNA Technologies, Zurich, Switzerland) was injected into fertilized oocytes (n = 250) to induce the complete Mup gene cluster deletion (Fig. 1a). Embryos were transferred into C57BL/6N foster mothers. Genotyping of pups was performed by PCR of gDNA using primers spanning the Cas9 cleavage sites (Fig. 1a). The first primer pair (MupF (5’-TCATGCTCCTGCAGTCTTC-3`) and MupwtR (5ʹ-GCCTCAGATCTTCTGTTACG-3ʹ); product size: 660 bp) identified WT animals. The second primer pair (MupF and MupkoR (5’CCCTCCAAACTGACTTTCTG-3’); product size: 370 bp) identified KO animals (Fig. 1b). The PCR product sizes were controlled by gel electrophoresis on a 1.5% agarose gel. A single male founder with a complete Mup cluster deletion was identified, verified using Sanger sequencing (Microsynth AG, Balgach, Switzerland), and used for breeding of the F1 generation. A heterozygous intercross was performed to generate homozygous offspring.

Male and female homozygous KO and WT mice were identified by PCR and confirmed by Sanger sequencing as described above. Twelve animals from each genotype/sex combination were selected to result in four experimental groups: mWT, fWT, mKO, and fKO.

The experimental animals were kept in groups of two to four mice under specific pathogen free conditions at a 12 h light:12 h dark cycle, 55% humidity, and a constant room temperature of 22 °C at the ETH Phenomics Center (Zurich, Switzerland). Animals had ad libitum access to water and a standard chow diet (3430 Haltung Standard, Kliba Nafag, 16.1 MJ/kg gross energy, 13.2 MJ/kg metabolizable energy, 18.5% crude protein, 4.5% crude fat, 38% starch; Granovit AG Kaiseraugst, Switzerland). Each randomization carried out in this study was computed with the publicly available random number generator (https://www.random.org). We did not define exclusion criteria for the animals prior to the experiment. However, one mKO mouse was found dead before the beginning of the study at the age of twelve weeks and could therefore not be included in the study. The KO mice can be made available upon request.

### Metabolic phenotyping

At 12 weeks of age, individual BW was determined. Subsequently, a subset of the experimental mice (mWT = 6, mKO = 5, fWT = 6, fKO = 6) were single-housed in a Phenomaster (TSE Systems GmbH, Bad Homburg, Germany) for two days of acclimatization followed by two days of data collection. Cage position of the individual animals within the Phenomaster instrument was randomized. Individual respiratory parameters, physical activity, and nutritional behavior were automatically monitored in intervals of ten minutes. Individual gas exchange was automatically calculated by comparison to an internal reference cage via the manufacturer’s phenotyping software (Version 5.6.3, TSE Systems GmbH). The phenotyping unit maintained a continuous airflow of 0.3 l/min during measurements. Food and water were refilled when necessary. Indirect calorimetry data was adjusted for individually calculated metabolic BW (lean mass + 0.2 × total body fat mass) to normalize for body composition-dependent metabolic turnover. Lean and total body fat mass were determined as described below. Physical activity was quantified by monitoring of total movements in the horizontal (X.T) and vertical planes (Y.T) via sensor frames equipped with infrared light beams surrounding the home cage.

### Body composition analysis

Mice (mWT = 12, mKO = 11, fWT = 12, fKO = 12) were fasted for a period of 4 h. Body composition was measured following a random order. After introducing full anesthesia by inhaling 2.5% isoflurane (≈ 5 min), mice were positioned along their longitudinal axis in a microCT scanner (VivaCT80, Scanco Medical AG, Brüttisellen, Switzerland) to assess adipose tissue volume in the region of the lumbar vertebrae L1–L5. After a two-dimensional pre-scan to define boundaries of L1–L5, the microCT scan was performed using the following settings: 117 mA current, 45 kVp voltage, 250 ms integration time, 300 projections per 180°, 216 slices in 4 stacks, 16.848 mm field of view, 13 min total scan time. Images were reconstructed at a nominal istotropic resolution of 78 µm. The VWAT and SWAT volumes were computed based on the algorithm of Judex et al.^59^ using Image Processing Language (IPL; Scanco Medical AG). Briefly, the muscular abdominal wall was manually defined in the reconstructed three-dimensional microCT images and a Gaussian filter was applied to reduce noise. Next, a full body mask was generated (threshold: 7–100% grey level) and overlaid with the previously defined region of the muscular abdominal wall. Pixels in the inner abdominal region within the range of 7–12.5% grey level were considered as VWAT, while pixels outside of the abdominal wall and within the given range were considered as SWAT. The VWAT and SWAT volumes were normalized to total volume (L1–L5). The VWAT volume in % related to BW was considered as estimate for the total body fat mass^59^ used in the equation for calculating the metabolic weight described above. The microCT data was validated via correlation analysis of VWAT proportions with GWAT mass.

### Plasma collection and tissue harvesting

After exiting the microCT, mice (n = 6 per group) were immediately euthanized by cardiac puncture. Whole blood was collected into EDTA tubes and centrifuged (1200×*g*, 10 min, 4 °C). Plasma was collected and snap frozen in liquid nitrogen. Following the confirmation of death by cervical dislocation, mice were opened longitudinally to harvest and weigh liver and GWAT. Prior to statistical analyses, tissue weights were normalized to BW. The liver was snap frozen and stored at − 80 °C until further analyses.

### Analyses of metabolic markers in plasma and liver

Leptin, adiponectin, FFA, insulin, glucose, and testosterone were quantified in EDTA plasma using commercially available ELISA kits (Crystal Chem, Chicago, IL, USA; FFA: Abnova Corp., Walnut, CA, USA) according to the manufacturer's instructions. Due to inaccurate measurements of the FFA ELISA kit caused by hemolysis, FFA values from three plasma samples (mKO: 1; fWT: 2) had to be excluded from the data set for this biomarker. The TG and cholesterol plasma concentrations were measured by enzymatic assays (Analyticon Biotechnologies AG, Lichtenfels, Germany) according to the manufacturer's protocol. To determine hepatic TG and cholesterol content, frozen liver tissue (~ 100 mg) was homogenized in 2 ml isopropanol using a dounce homogenizer (tight pestle: clearance of 0.0005–0.0025 inch, 15 strokes) and incubated for 10 min while shaking at RT. Subsequently, liver homogenates were centrifuged (3000 rpm, 10 min, RT) and the collected supernatant was applied in the enzymatic assays. The obtained values were normalized to initial tissue input.

### Western Blot analysis in liver and urine

Fresh liver samples (~ 100 mg) were homogenized using a dounce homogenizer (loose pestle: clearance of 0.0025–0.0055 inch, 5–7 strokes). Cytoplasmatic proteins were isolated by adding 500 µl ice-cold pre-extraction buffer (Nuclear Extraction Kit, Abcam, Cambridge, UK). After 15 min of incubation on ice, liver homogenates were centrifuged (12,000 rpm, 10 min, 4 °C) and the supernatant was transferred into a new tube. The cytoplasmatic fractions were stored at − 80 °C.

At twelve weeks of age, hydrophobic sand (Zoonlab, Castrop Rauxel, Germany) was applied to the home cages to collect spot urine. To account for individual dilution, urine samples were normalized to urinary creatinine concentration, determined via a colorimetric assay (abcam) after deproteinization by 4% perchloric acid.

The samples (liver: 20 μg protein; urine: 1.5 μg creatinine equivalents) were loaded onto a stain-free polyacrylamide (4–20%) gel (Biorad, Cressier, Switzerland) and separated via electrophoresis. After gel activation, proteins were transferred onto PVDF membranes. Immunoblotting was performed using anti-Mup (1:500), and anti-mouse IgG (1:10,000) (both: Santa Cruz, Heidelberg, Germany). Protein bands were detected via enhanced chemiluminescence in a ChemiDoc MP (BioRad).

### Hepatic Isolation of DNA and RNA

The frozen liver tissue (~ 30 mg) was homogenized using ceramic beads (Roche, Basel, Switzerland) following standard procedures of mechanical tissue disruption. The gDNA and total RNA were extracted using the All Prep RNA/DNA Kit (Qiagen, Hilden, Germany). The purity and quantity (based on the 260:280 nm and 260:230 nm ratios) of the isolated gDNA and RNA were analyzed using a NanoDrop (peqLab, Erlangen, Germany). The RNA quality number (RQN) was determined using the fragment analyzer instrument (Agilent Technologies, Santa Clara, CA, USA). The RQN was ≥ 8.4 for all samples.

### Hepatic gene expression analysis

Sequencing libraries were prepared according to the manufacturer’s instructions of the TruSeq Stranded mRNA library prep kit (Illumina, San Diego, CA, USA) using an input of 300 ng total RNA per sample (n = 6 per group). Quality controlled libraries were sequenced in a single multiplex on a NovaSeq instrument (Illumina) in 100 bp single-end read mode. Overall, the sequencing depth was about 25 million reads per sample. Quality-based adapter trimming was executed using fastp^60^. Filtered and trimmed sequences were aligned to the mouse reference genome (GRCm38.p6) using STAR^61^ with a global alignment rate of 90%. Raw read counts were calculated with the featureCounts option of the Rsubread package^62^. For biological reliability, genes with low expression levels were excluded according to the method described by Chen et al.^63^. Accordingly, genes with less than 0.66 counts per million (CPM) in at least six out of the total 24 libraries were removed. The analysis of DEG was performed using edgeR with a cut-off *p* < 0.01 for a true DEG^64^. The obtained DEG data sets were submitted to functional annotation by performing comparative analysis of mKO and fKO mice using the Ingenuity Pathway Analysis (IPA; http://www.ingenuity.com/index.html) tool (Qiagen).

### Hepatic DNA methylation analysis

Digestion of 500 ng of gDNA per sample (n = 6 per group) was performed using MspI (Tango Buffer; 6 h, 37 °C; Thermo Scientific, Zurich, Switzerland), followed by nucleotide end-repair and A-tailing (Klenow Fragment exo- with dCTPs, dGTPs, and dATPs in Tango Buffer; 40 min, 37 °C; Thermo Scientific) and heat inactivation (15 min, 75 °C). Next, methylated adapters (Next EM-seq Adaptors; NEB E7165A, New England Biolabs, Frankfurt am Main, Germany) were ligated to the A-tailed DNA fragments using T4 DNA ligase (HC; Thermo Scientific) overnight at 4 °C. Subsequently, bisulfite conversion of the libraries was performed followed by cleaning according to the manufacturer’s instructions (ZYMO EZ-96 DNA Methylation-DirectTM MagPrep, Zymo Research, Freiburg, Germany). Finally, the bisulfite converted libraries were amplified (10 cycles) using an uracil stalling free polymerase (Kapa Uracil + 2 × MasterMix, Roche) and purified (0.8 × SPRIselect; Agencourt, Beckman Coulter, Krefeld, Germany). Then the libraries were analysed on a TapeStation (Agilent), quantified (NEBNext Library Quantification Kit E7630), and sequenced on a NextSeq500 (HighOutput, 150 cycles, Illumina).

FastQ files were trimmed to filter out low-quality reads and adapters using Trim Galore v0.6.4 (https://www.bioinformatics.babraham.ac.uk/projects/trim_galore/). Remaining sequencing reads were mapped to the reference mouse genome assembly GRCm38 using Bismark v0.22.3 with default parameters in paired-end mode^65^. The CpGs were extracted using Bismark methylation extractor v0.22.3 in paired-end mode and were subsequently analyzed for differential methylation using the R (v4.0.4) package edgeR v3.32.1^64^. With the function nearestTSS from edgeR, the CpGs were annotated to the closest gene transcriptional start site (TSS). For biological reliability, consistently methylated or non-methylated CpGs were filtered out for each sex. Similarly, CpGs with low coverage (≤ 5 total counts in at least three samples) were removed for each combination of genotype and sex. The analysis of differentially methylated regions (DMR) was restricted to the promoter region, defined as the regions located 2 kb up and 2 kb downstream of the TSS. Gene promoters were split into CpG island (CGI) and non-CGI based on their overlaps with CGIs using the R package annotatr v3.13^66^. A DMR was defined by ≥ 5% methylation difference in the sex-specific KO vs. WT comparisons and *p* < 0.05. One mKO library failed sequencing and therefore had to be excluded from subsequent DMR analysis. Mouse age was estimated using the epigenetic clock (https://github.com/EpigenomeClock/MouseEpigeneticClock) based on their RRBS profile^24^.

### Statistical analysis

Data was analyzed in R Version 3.6.2 (R core Team, 2019). Shapiro-Wilk test was performed to control for normal distribution. For normally distributed data of BW, body composition (degrees of freedom: mKO vs. mWT = 21, fKO vs. fWT = 22), organ weights (degrees of freedom: mKO vs. mWT = 10, fKO vs. fWT = 10), and all plasma biomarkers (degrees of freedom: mKO vs. mWT = 10, fKO vs. fWT = 10; FFA: mKO vs. mWT = 9, fKO vs. fWT = 8), a sex-specific two-tailed t-test was applied. For some parameters, non-normal data distribution was confirmed (males: SWAT, testosterone, total activity, liver proportion; females: VWAT, SWAT, glucose, insulin, EE, liver proportion). For these parameters, Mann-Whitney U test was performed as non-parametric alternative. Variance homogeneity of the data was controlled with Levene test. Parameters displayed sex-specific equal variances between genotypes throughout the analyzed parameters except for plasma cholesterol in males. In this case, non-equal variance between experimental groups was accounted for by performing Welch t-test for statistical comparison. Potential effects of birth date, cage, and batch were excluded by computing likelihood ratio tests. For respiratory parameters, a linear model was fitted per sex and genotype. Effects throughout the measurement period were analyzed accounting for repeated measurements. Sex-specific genotype effects at individual timepoints were calculated using two-tailed t-test (degrees of freedom: mKO vs. mWT = 9, fKO vs. fWT = 10). Data are presented as boxplots with Spear style whiskers, indicating the minimum and maximum values. A *p* < 0.05 was considered as statistically significant.

## Supplementary Information

Supplementary Information 1.

Supplementary Information 2.

Supplementary Information 3.

## Data Availability

The Fastq files from RNAseq and RRBS experiment were deposited in NCBI's Gene Expression Omnibus and are accessible through GEO Series accession numbers GSE188156 and GSE188157 and in combination through GSE188158. All other data are included within the article, source data, and supplementary data can be made available upon request.
