# Supplementary material for: The major urinary protein gene cluster knockout mouse as a novel model for translational metabolism research
Source: Sci Rep. 2022 Aug 1;12:13161. doi: 10.1038/s41598-022-17195-y (PMC9343454; doi:10.1038/s41598-022-17195-y)
Supplement: Supplementary file 3 — Supplementary Information 3. [file 41598_2022_17195_MOESM3_ESM.docx]

*Supplementary data for*

***The major urinary protein gene cluster knockout mouse as a novel model for translational metabolism research***

*Sarah Greve, Gisela A. Kuhn, Mara D. Saenz-de-Juano, Adhideb Ghosh, Ferdinand von Meyenn, Katrin Giller*

**Supplementary Tables** (separate files)

**Dataset 1:** Differential expression analysis of RNAseq data.

**Dataset 2:** Differential expression analysis of RRBS data.

**Supplementary Figures**


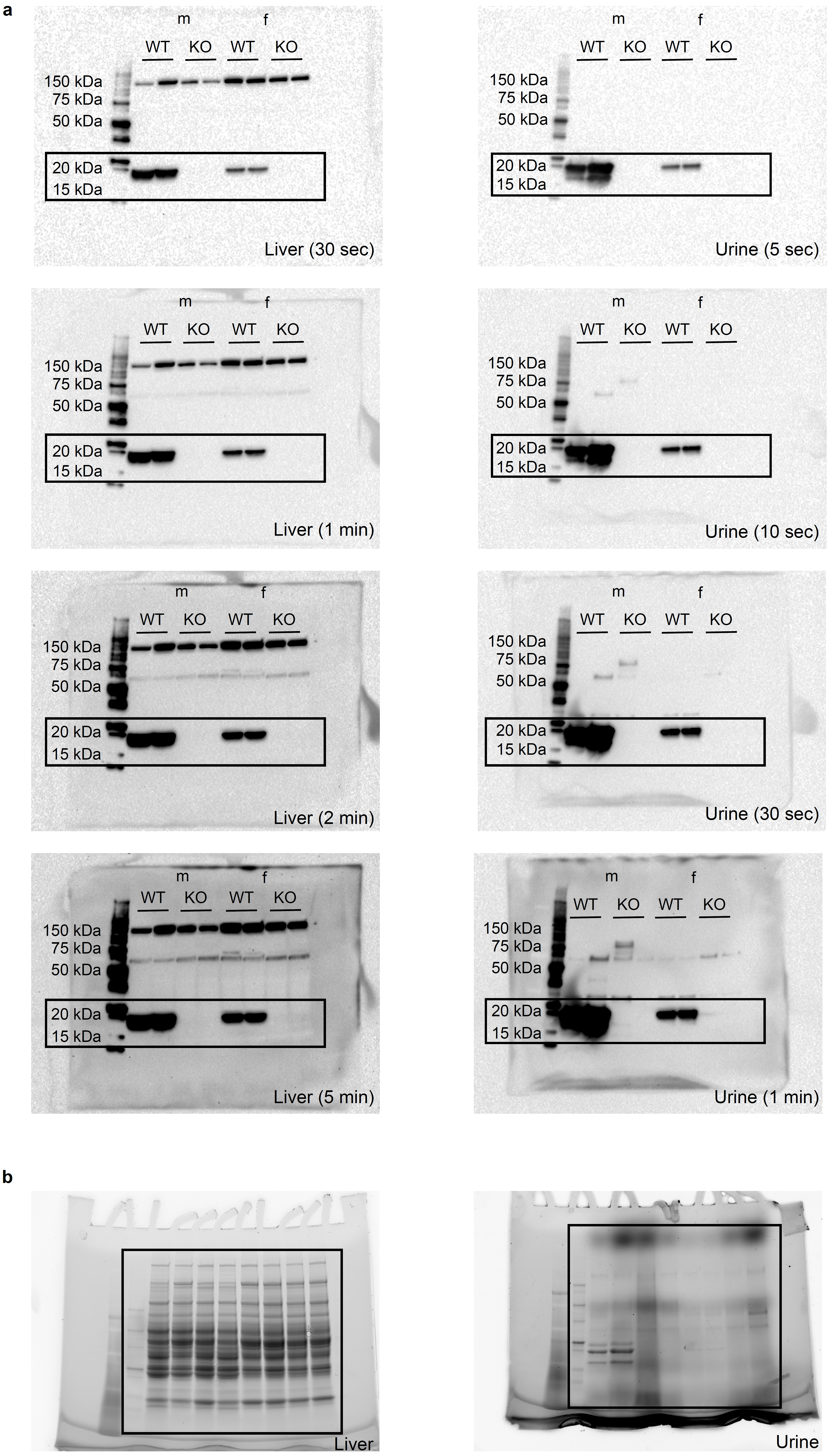


**Supplementary Fig. 1 Multiple exposure images from** original blots of representative liver and urine samples in male (m) and female (f) Mup knockout (KO) and wildtype (WT) mice. Individual exposure time is indicated in brackets. **b** Respective loading controls onto stain-free polyacrylamide gels. Black rectangles represent the cropped image in Fig. 1 of the main manuscript.

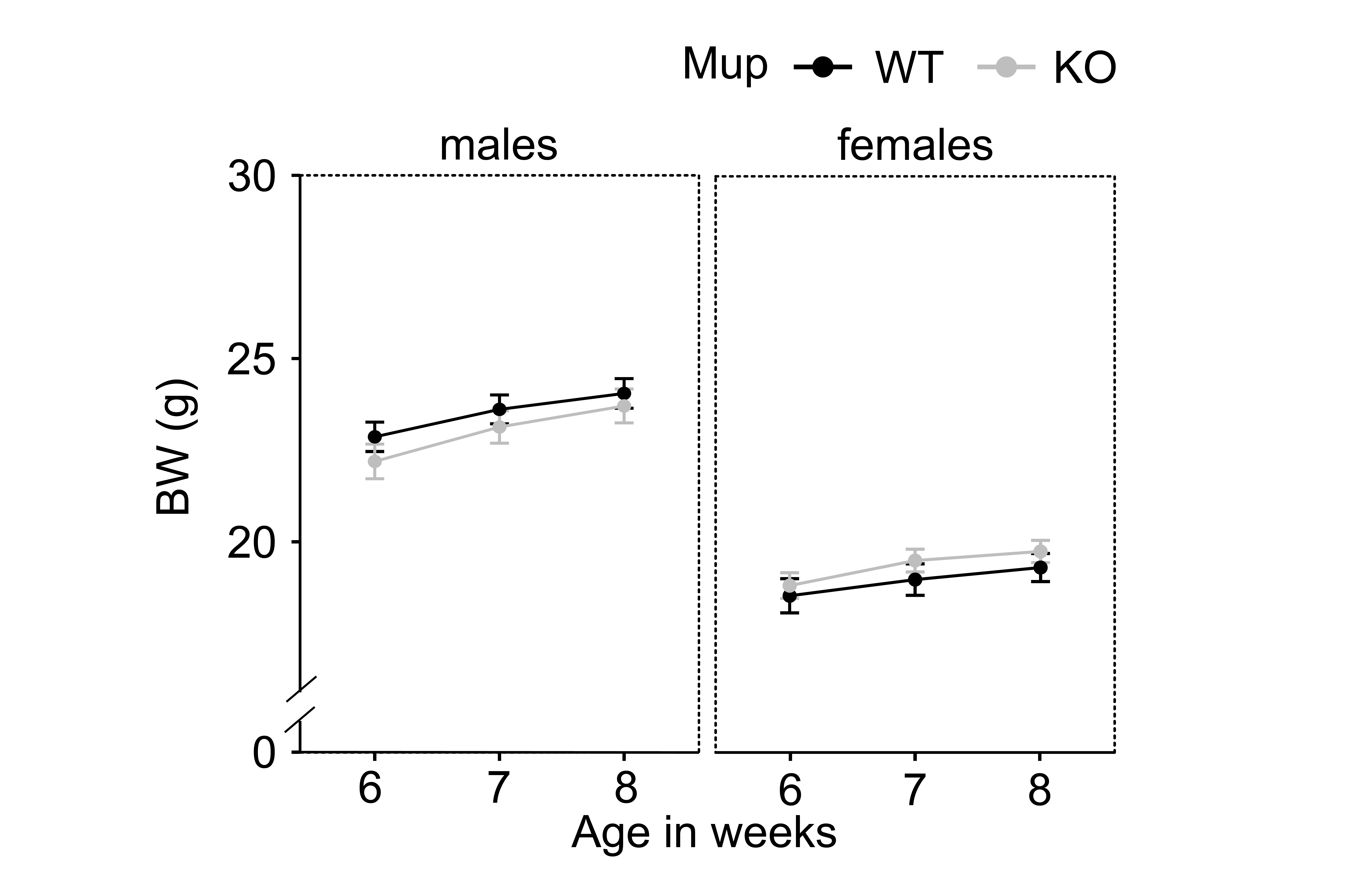


**Supplementary Fig. 2** Weekly body weight (BW) development in young mice prior to testosterone induced substantial production of major urinary protein (Mup) in male (m) and female (f) knockout (KO) and wildtype (WT) mice (mWT = 16, mKO = 16, fWT = 16, fKO = 16). Results are presented as mean ± SEM.


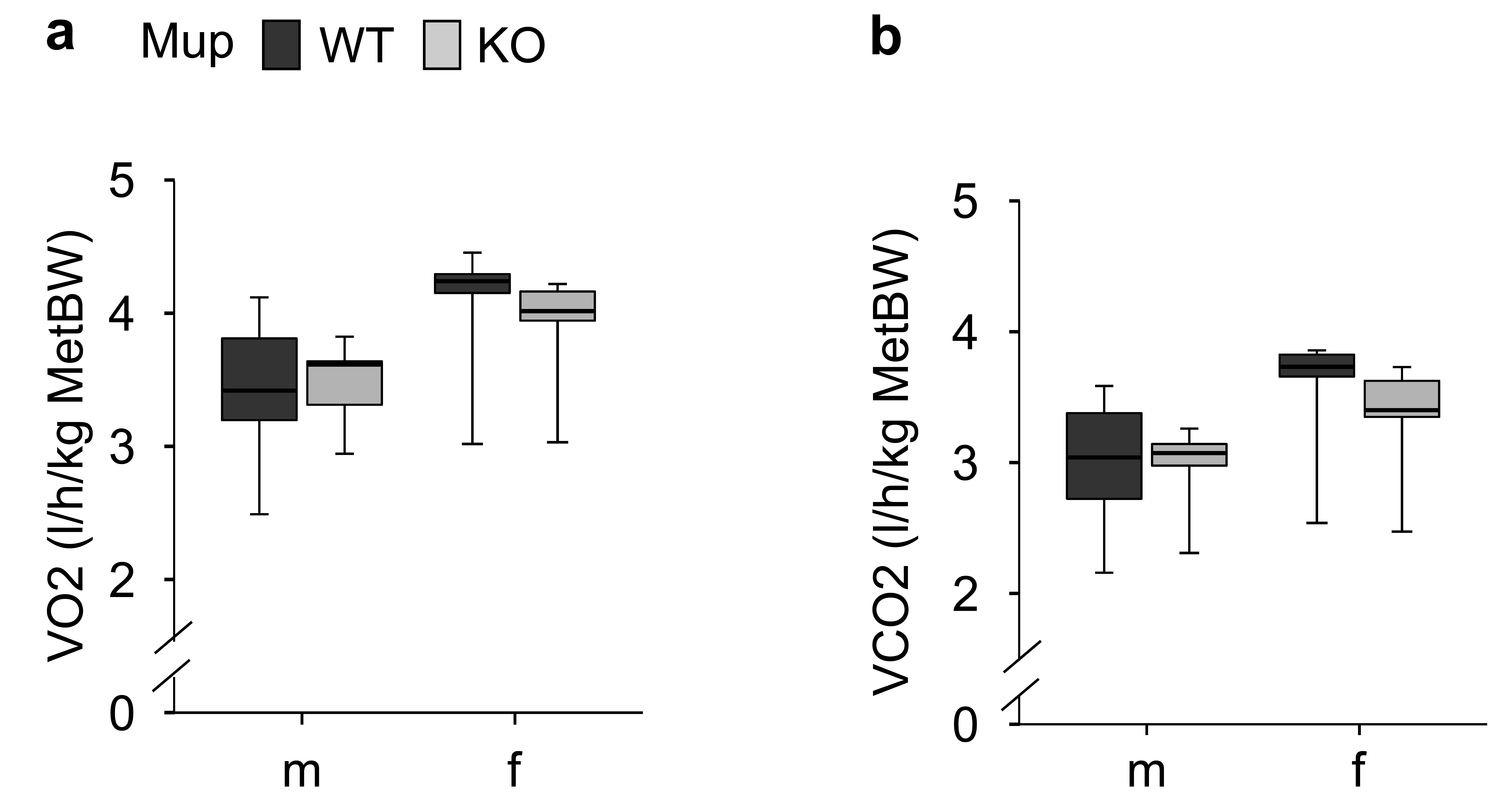


**Supplementary Fig. 3 (**a**)** Sex specific mean oxygen uptake (VO_2_) and (**b**) carbon dioxide production (VCO_2_) according to genotype monitored over 48 hours in the Phenomaster (mWT = 6, mKO = 5, fWT = 6, fKO = 6). Data was adjusted to metabolic body weight (MetBW; lean mass+0.2×fat mass). Data is presented as Spear style boxplots, where the whiskers indicate the minimum and maximum value, lower and upper hinges correspond to the upper and lower quartiles, and center line to the median.


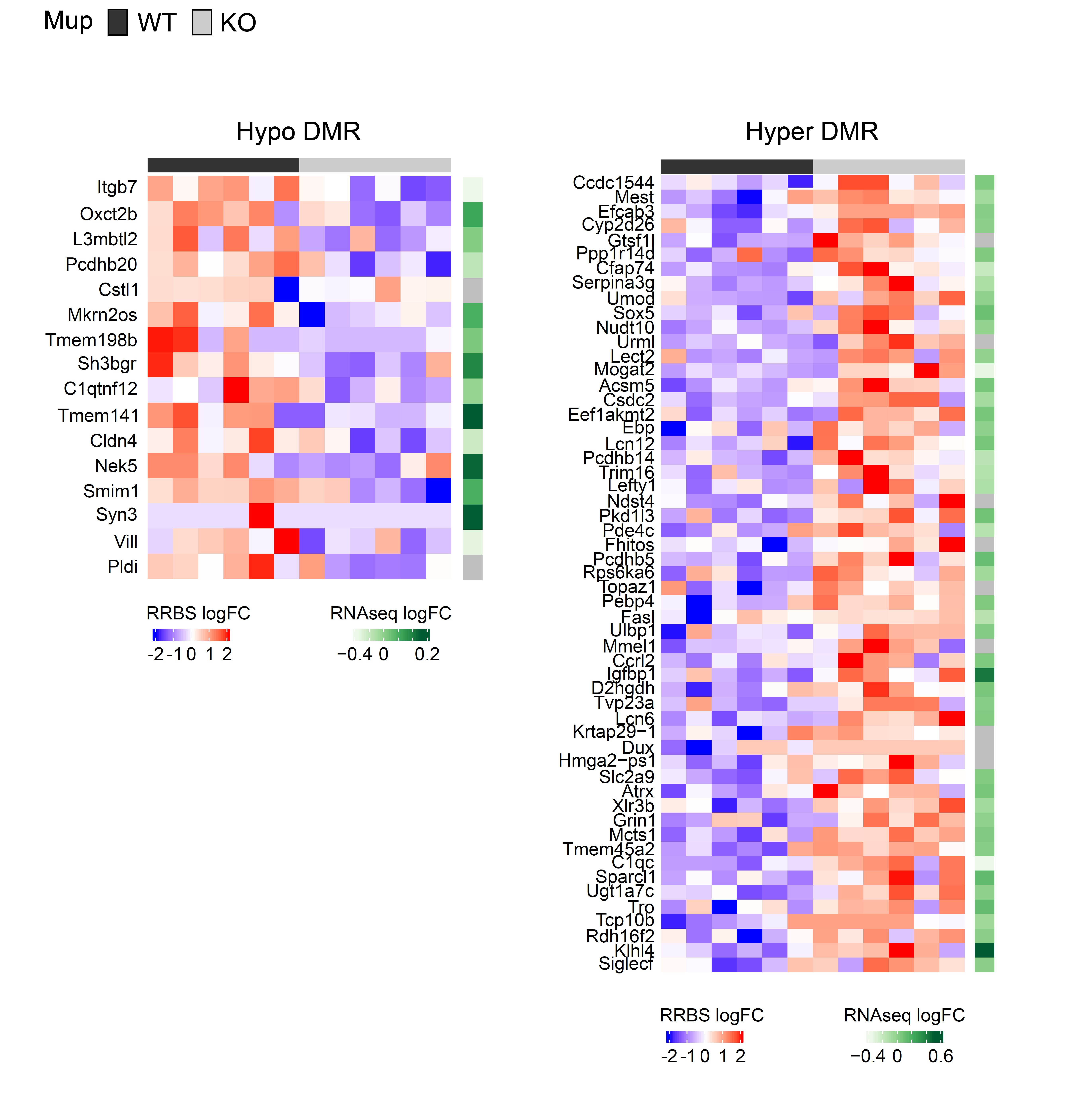


**Supplementary Fig. 4** Heatmap showing the significant (*p* < 0.05) hypo- and hypermethylated differentially methylated regions (DMR) with > 5% methylation difference in female knockout (KO) mice and wildtype (WT) mice (n = 6). Color gradient corresponds to individual DMR methylation status (red: hypermethylated, blue: hypomethylated) per sample and corresponding gene expression level retrieved from KO vs. WT differential gene expression analysis (green: upregulated, grey: downregulated)
